# Supplementary material for: Curated and harmonised transcriptomics datasets of interstitial lung diseases
Source: Data Brief. 2025 Oct 14;63:112139. doi: 10.1016/j.dib.2025.112139 (PMC12581653; doi:10.1016/j.dib.2025.112139)
Supplement: Supplementary file 1 [file mmc1.zip › Supplementary_material/RNA-seq/fastQC reports/GSE184316/SRR15926785_fastqc.html]

SRR15926785.fastq.gz FastQC Report 

FastQC Report

Mon 5 Feb 2024  
SRR15926785.fastq.gz

## Summary

- Basic Statistics
- Per base sequence quality
- Per sequence quality scores
- Per base sequence content
- Per sequence GC content
- Per base N content
- Sequence Length Distribution
- Sequence Duplication Levels
- Overrepresented sequences
- Adapter Content

## Basic Statistics

| Measure | Value |
| --- | --- |
| Filename | SRR15926785.fastq.gz |
| File type | Conventional base calls |
| Encoding | Sanger / Illumina 1.9 |
| Total Sequences | 7429581 |
| Sequences flagged as poor quality | 0 |
| Sequence length | 25-380 |
| %GC | 50 |

## Per base sequence quality

## Per sequence quality scores

## Per base sequence content

## Per sequence GC content

## Per base N content

## Sequence Length Distribution

## Sequence Duplication Levels

## Overrepresented sequences

| Sequence | Count | Percentage | Possible Source |
| --- | --- | --- | --- |
| CAGTGGGGCCACGAGCTGAGTGCGTCCTGTCACTCCACTCCCATGTCCCT | 75997 | 1.022897522753975 | No Hit |
| AGGCTACACACTGAGGACTCTGTTCCTCCCCTTTCCGCCTAGGGGAAAGT | 48082 | 0.6471697394509865 | No Hit |
| CAATCCACTTGATCCCAACTCATCTCTCATTTATTTCGGCTTCTTTTATT | 42314 | 0.5695341365818611 | No Hit |
| TGATGACACCCACCGCAACTGTCTGTCTCATATCACGAACAGCAAAGCGA | 37934 | 0.5105806101313116 | No Hit |
| GTCCACGTCACACTTCATGATGGAGTTGAAGGTAGTTTCGTGGATGCCAC | 29428 | 0.396092323375975 | No Hit |
| TGATATGGTTCCTGGCAAGCCCATGTGTGTTGAGAGCTTCTCAGACTATC | 28688 | 0.38613213854186396 | No Hit |
| GGCATCAAAAGTGATGGACTGCCCATTGCTGGAGAAGACCTTCTCTCCTA | 28279 | 0.38062711746463224 | No Hit |
| GCCGACAAGACCAACGTCAAGGCCGCCTGGGGTAAGGTCGGCGCGCACGC | 28044 | 0.3774640857943402 | No Hit |
| TACCACTGCCACCTTCTCCATCGGCTCCACTGGCCTCGTGGTGTATGACT | 27083 | 0.3645293052192311 | No Hit |
| GGCAATGAGCGGTTCCGCTGCCCTGAGGCACTCTTCCAGCCTTCCTTCCT | 26498 | 0.3566553753165892 | No Hit |
| CCCTGGCTGCTACAGGAGAATAGCAGACAGGTATAGTTAAGAAACAACTT | 21203 | 0.28538621491575367 | No Hit |
| GGGAAGCGGGGAATGTCTACGTGCGTATGCACGTGGCACTCTCTGCCCGA | 20361 | 0.27405313973964346 | No Hit |
| AGCTGCTAAGTGCTGTGTTGTCGTTCCCCCTGCTTAAAATAAAGTTGTTT | 18068 | 0.2431900264631343 | No Hit |
| TTACAAAAGCAGAATAAAAGCGAAAAGAAATGAAAATGTTACACTACATT | 17311 | 0.23300102657202335 | No Hit |
| AGACATCAAATCCTGCAGACAAGGGGAGCCCTCAGTCTGCAGGGCTCCAT | 17004 | 0.22886889583679082 | No Hit |
| CTTCATGATGTAGCAGCAGGTGCCAGGGGCTGGCTTGTAGGCGATCAGCA | 16847 | 0.226755721486851 | No Hit |
| CTCCGCCGCAGACGCCGCCGCGATGCGCTACGTCGCCTCCTACCTGCTGG | 14992 | 0.20178796085539683 | No Hit |
| CAATGGGGTACTTCAGGGTCAGGATGCCACGCTTGCTCTGGGCCTCGTCG | 13979 | 0.18815327540005283 | No Hit |
| CAGCATGTTCCCTCTCCTCATGAGATTGGTGAAGAAAGTATTTGGCAAAG | 13501 | 0.18171953438558647 | No Hit |
| ACGTTTACCTGTCCATGTCTTACTACTTTGACCGCGATGATGTGGCTTTG | 13236 | 0.17815271143823588 | No Hit |
| AACAAGCACAAAGCCAAGAAAACCAACGATGCCAGATACTGAATTTCTAT | 12847 | 0.17291688454570991 | No Hit |
| GGCAATGAGCGGTTCCGGTGTCCGGAGGCGCTGTTCCAGCCTTCCTTCCT | 12800 | 0.17228427821165151 | No Hit |
| GTCCACGTCACACTTCATGATGGAGTTGAAGGTGGTCTCGTGGATGCCGC | 12331 | 0.1659716745803027 | No Hit |
| CAGCAGTTTACACCTGCTAGGGTGTTCAAAGGTCAGTGCTATAGAAATTC | 11950 | 0.16084352536165902 | No Hit |
| TGCCATATCCACATGGAAAATCTCATCACCATCAAAGTCAAACATAAACT | 11858 | 0.15960523211201277 | No Hit |
| CGGCGAGAGCATGACCGATGGATTCCAGTTCGAGTATGGCGGCCAGGGCT | 11672 | 0.1571017261942497 | No Hit |
| TCAGGAGACGCTCGTAGCCCTCGCGCTTCTCCTCGGCCAGTTCGCGGAAG | 11529 | 0.15517698777360392 | No Hit |
| TCAGGAGACGCTCGTAGCCCTCGCGCTTCTCCTCGGCCAATTCGCGGAAG | 11456 | 0.1541944289994281 | No Hit |
| CTCTCTTTCTGGCCTGGAGGCTATCCAGCGTACTCCAAAGATTCAGGTTT | 11396 | 0.15338684644531098 | No Hit |
| CGAAGCGGTTGTACTTGCGGATGTAGTGCAGATAGTCTCGGCGGATGACA | 11222 | 0.15104485703837134 | No Hit |
| ACAAGAAGTACATCAAAGATTACATGAAATCAATCAAAGGGAAACTTGAA | 10985 | 0.14785490594960873 | No Hit |
| CGTGAATACCAAGACCTGCTCAATGTTAAGATGGCCCTTGACATTGAGAT | 10649 | 0.14333244364655287 | No Hit |
| ACACTGTTCTTGTAGATGGCTGCTCTAAAAAGACAAATGAATGGGGAAAG | 10427 | 0.14034438819631956 | No Hit |
| AGGTCTTGGTGGTGGGGAAGGACAGGAACATCCTCTCCAGGGCCTCCGCA | 9841 | 0.13245699858444238 | No Hit |
| CCAAGATCTTCTTGATGTCCTTGGCGCTGGGGGAGGAGTTGCCCCCTAGG | 9822 | 0.13220126410897196 | No Hit |
| CGATCAGCACGGGCTCGTACTCTGCCACAAACTGATCACACTGCTTCTGG | 9725 | 0.13089567231314927 | No Hit |
| GGCAGACAAACCAGACATGGGGGAAATCGCCAGCTTCGATAAGGCCAAGC | 9640 | 0.12975159702815006 | No Hit |
| AGACCACCAGCAGCCTGCCCAGGGCCTCACCACCAACTTC | 9309 | 0.12529643327127063 | No Hit |
| TCTTGGGTTATAATCATTGGCAATGTTGTAATCAACAACCCATGTTATTT | 9244 | 0.12442155217097708 | No Hit |
| CTGACAGTGTATCTAGCTTTGGAAAACACAGGGGTCTGCCCTGTGAGCTG | 8866 | 0.11933378208003924 | No Hit |
| GACCGAATTCCCCCTGCGCATGCGGGACTGGCTCAAGAACGTCCTGGTCA | 8672 | 0.11672259848839389 | No Hit |
| CAATAAATAGGACACTTCTGCCAAAGGAACTCGTGCACAGCACCACAGAA | 8433 | 0.11350572798116071 | No Hit |
| TCGCTCGTACTCGTGCGCCTCGCTTCGCTTTTCCTCCGCAACCATGTCTG | 8347 | 0.11234819298692618 | No Hit |
| TCTTCACCCGCAGCTTCTGCTTCTCAGTCAGAAGGTTGTTGTCCTCATCC | 8325 | 0.1120520793837499 | No Hit |
| GCGTCTCTGTCTTCTTCAGTTTCGACTTATCGAATTTCTCGATCTCAGCC | 8204 | 0.11042345456628039 | No Hit |
| TGTGGATGAATCTTGAACTGAGTTCCACTTGTAAACTTCTTGTTTCTTGT | 8194 | 0.11028885747392753 | No Hit |
| GAAACCCAGACACATAGCAATTCAGGAAATTTGACTTTCCATTCTCTGCT | 8058 | 0.10845833701792874 | No Hit |
| CCAAAGGTGCAATATCAAGGAAGGGCAGGCGTGATGGCTTATTTGTTTTG | 8006 | 0.10775843213769391 | No Hit |
| GGAGGATGGTTGCACGAAACACACTGGGGAATGGAGCAAAACAGTCTTTG | 7967 | 0.10723350347751777 | No Hit |
| GGAAGACAGTGTGGCGATTCCTCAGGGATCTAGAACTAGAAATACCATTG | 7862 | 0.10582023400781282 | No Hit |
| CGAGCCACAGCTCTGCCAGTACCCCAAGACTCAGCACTAGTCTGATGACC | 7813 | 0.10516070825528384 | No Hit |
| GACGAGTCAGAAAGGTCACGTCCTGCTCTTGGTCCTTGTCAGTGCCATGT | 7803 | 0.105026111162931 | No Hit |
| CCACAACTTCCTTCTTCTCCTTTAAGTCCTTGGTGGTGATTTCGGAGCTG | 7568 | 0.10186307949263894 | No Hit |

## Adapter Content

Produced by FastQC (version 0.11.7)
